# Aurochs roamed along the SW coast of Andalusia (Spain) during Late Pleistocene

**DOI:** 10.1038/s41598-022-14137-6

**Published:** 2022-06-14

**Authors:** Carlos Neto de Carvalho, Fernando Muñiz, Luis M. Cáceres, Zain Belaústegui, Joaquín Rodríguez-Vidal, João Belo, Noel Moreira, Mário Cachão, Pedro P. Cunha, Silvério Figueiredo, José María Galán, Yilu Zhang, Paula Gómez, Antonio Toscano, Francisco Ruiz, Samuel Ramírez-Cruzado, Francisco Giles-Guzmán, Geraldine Finlayson, Stewart Finlayson, Clive Finlayson

**Affiliations:** 1 Naturtejo UNESCO Global Geopark, Geology Office of the Municipality of Idanha-a-Nova, Idanha a Nova, Portugal; 2 Instituto D. Luiz, University of Lisbon, Lisbon, Portugal; 3 Departamento de Cristalografía, Mineralogía y Química Agrícola, Universidad de Sevilla, Sevilla, Spain; 4 Departamento de Ciencias de la Tierra, Universidad de Huelva, Huelva, Spain; 5 Departament de Dinàmica de la Terra i de l’Oceà, Facultat de Ciències de la Terra, Institut de Recerca de la Biodiversitat (IRBio), Universitat de Barcelona (UB), Barcelona, Spain; 6 Geosciences Center, FlyGIS-UAV Surveys, University of Coimbra, Coimbra, Portugal; 7 Instituto de Investigação e Formação Avançada, Institute of Earth Sciences (ICT)-Pole of Évora, University of Évora, Évora, Portugal; 8 Department of Geology, Faculty of Sciences, University of Lisbon, 1749-016 Lisboa, Portugal; 9 Department of Earth Sciences, MARE-Marine and Environmental Sciences Centre, University of Coimbra, Coimbra, Portugal; 10 Department of Archeology, Conservation and Heritage, CGeo-UC, Polytechnical Institute of Tomar, Tomar, Portugal; 11 Centro Português de Geo-História e Pré-História, São Caetano, Portugal; 12 Centro Administrativo del Acebuche, Parque Nacional de Doñana, Matalascañas, Spain; 13 Academy of Natural Resources of Henan, Zhengzhou, China; 14 The Gibraltar National Museum, Gibraltar, UK; 15 Institute of Life and Earth Sciences, University of Gibraltar, Gibraltar, UK; 16 Department of Life Sciences, Liverpool John Moores University, Liverpool, UK; 17 Department of Anthropology, University of Toronto, Scarborough Campus, Toronto, Canada

**Keywords:** Palaeoecology, Geomorphology, Palaeontology, Sedimentology

## Abstract

In the Iberian Peninsula the fossil record of artiodactyls spans over 53 million years. During the Pleistocene, wild cattle species such as *Bison* and especially *Bos* became common. In Late Pleistocene, the aurochs (*Bos primigenius*) was widespread and the only bovine living along the large river valleys of southern Iberia. Although commonly found in fossil sites and especially in cave bone assemblages, the trace fossil record of aurochs was known worldwide only from the Holocene. Large bovine and roe deer/caprine tracks were found in at least five horizons of the early Late Pleistocene (MIS 5) beach and eolian deposits of Cape Trafalgar (Cadiz Province, South of Spain). The large bovine tracks are formally described as *Bovinichnus uripeda* igen. *et i*sp. nov. and compared with the record of aurochs tracks, large red deer tracks and steppe bison biogeographical distribution in Iberia. Aurochs were the most likely producers of the newly described Trafalgar Trampled Surface (TTS) and some of the large artiodactyl tracks in the Matalascañas Trampled Surface, representing the oldest aurochs track record known. This new evidence, together with comparisons with the record of possible aurochs tracks in the Mid-Late Pleistocene coastal deposits from the Asperillo cliff section in Matalascañas (Huelva Province, SW Spain) and bone assemblages known in Gibraltar, point to a recurrent use of the coastal habitat by these large artiodactyls in SW Iberia.

## Introduction

The artiodactyls are even-toed ungulates belonging to a diversified group of large land mammals that can be found nowadays all over the world, except Antarctica. The oldest fossils of even-toed ungulates date back to the early Eocene and were found in Europe, Asia and North America^1^. The oldest bone remains known in Europe were found in the Iberian Peninsula and are dated from this period^2,3^. However, their record of living activities, known through the study of trace fossils, such as tracks or excrements, is much more scattered, and known only since the late Eocene to early Oligocene in North America and Europe, including Spain^4–7^ (see the recent attribution of late Paleocene tracks to possible basal artiodactyls or tapiroids^8^).

**Figure 1:**
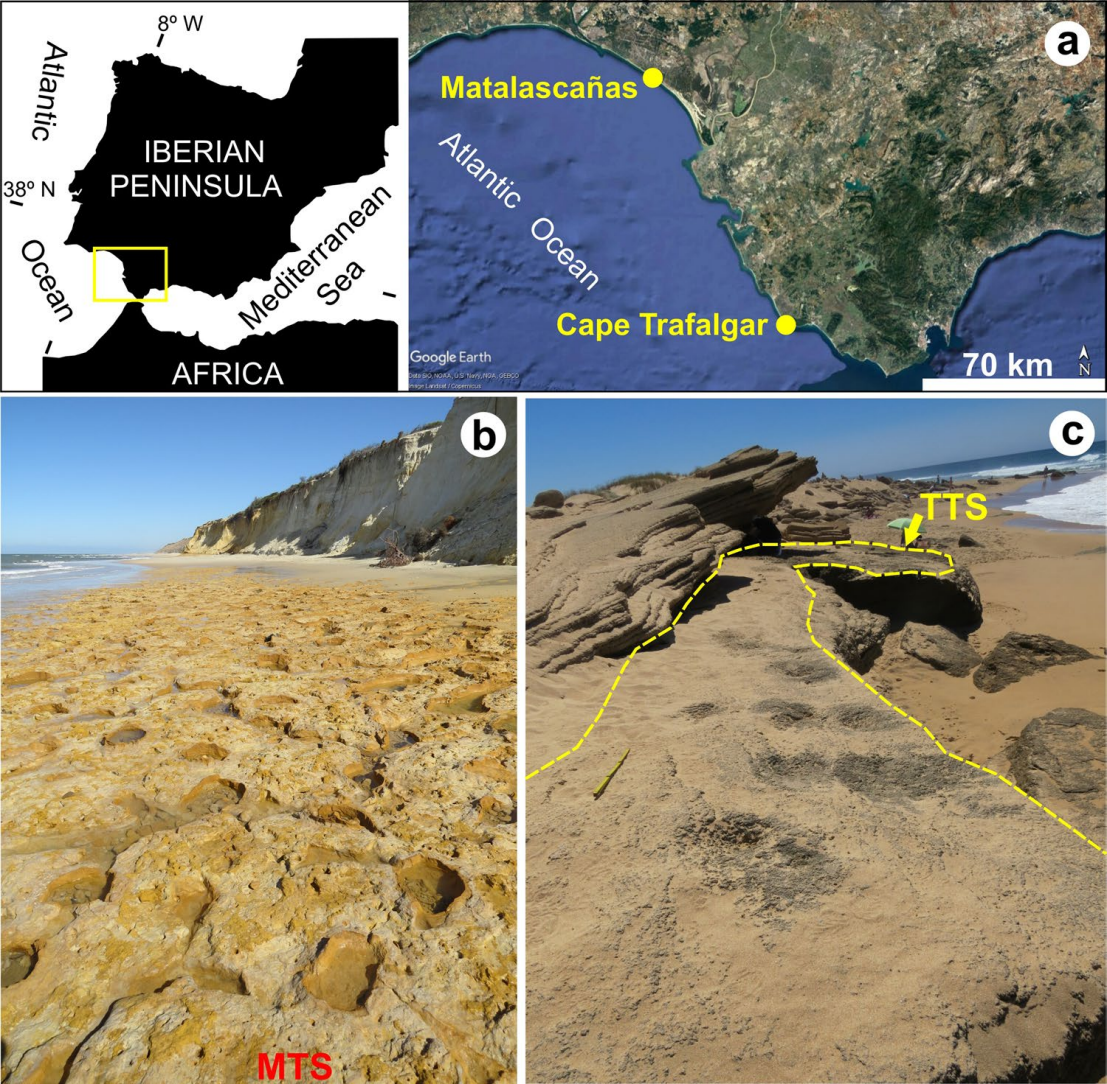
(**a**) Location of Cape Trafalgar and Matalascañas tracksites in SW Iberian Peninsula, Image adapted from GoogleEarth©; (**b**) The MTS at Matalascañas, Province of Huelva (SW Spain); (**c**) the new TTS in Cape Trafalgar (Province of Cadiz). Map in (**a**) (left) and modifications to the photograph in (**c**) have been drawn with CorelDraw 12 (https://www.coreldraw.com/la/).

Fossil tracks, when safely attributed to a specific producer, can be complementary to the osteological record, providing additional data on geographical distribution of species, their paleoecology and biological behaviour. The body fossil record of the bovine tribe is relatively common in Quaternary deposits, but rarely have tracks been attributed to these animals. In the Iberian Peninsula, artiodactyl tracks from Pleistocene beds were mostly attributed to goats, cervids, and wild boar^9–16^. Here we formally describe, for the first time, large artiodactyl tracks from two different sites in SW Spain ascribed to the early Late Pleistocene, safely attributed to aurochs (*Bos primigenius*). During the Late Pleistocene, the aurochs were widespread and the only wild cattle living along the large river valleys in southern Iberia. Artiodactyls are cloven-hoofed animals, which in most cases bear the body weight equally on two of their five toes: the third and fourth. We compare the morphology of very large cloven-hoofed tracks found during the study of Late Pleistocene eolianites in SW Iberia, with the ones of cervids and recent tracks made by *Bos taurus*. Although commonly found in fossil sites and especially in cave bone assemblages^17–19^, the trace fossil record of aurochs was only known from the Holocene of Great Britain, especially in tidal flat deposits from estuarine areas^20,21^. In SW Spain, the Cape Trafalgar and Asperillo cliff new tracksites (Fig. 1) provide an opportunity to endorse paleoecological interpretations regarding the recurrent use of the coastal habitats by these massive grazers during the Quaternary.

## The track fossil record of artiodactyls in the Iberian Peninsula and the presence of aurochs in the SW

In the Iberian Peninsula, the oldest cloven-hoofed tracks were identified in the Lower Miocene^22^. The first tracksites were described in the Late Pleistocene (MIS 3) of the Balearic Islands in Mallorca, Menorca and Formentera^9,11,23^, and attributed to the insular goat *Myotragus balearicus* Bate. Large cervid tracks were described in the Late Pleistocene eolianites of Gorliz (Basque coast, north Spain)^10^, in the Late Pleistocene (MIS 5–4) eolianites of SW Alentejo (Portugal)^12–14^, and in the Late Pleistocene of the Catalan Bay eolianite, in Gibraltar^24^. However, if the eolianites are confirmed to be MIS 3 in age, the more rounded cervid didactyl tracks from Gorliz could also be compared to those of the reindeer *Rangifer tarandus*, a species commonly found from the bone record in the northern Spain during the Pleniglacial^25–27^. Recently, the Matalascañas Trampled Surface (MTS) in the province of Huelva (SW Spain), dated from the early Late Pleistocene (MIS 5), was described showing abundant artiodactyl tracks which were attributed to wild boar, red deer and possibly aurochs^15,16^.

Aurochs tracks have been studied especially in coastal deposits in England and Wales associated with early Mesolithic to early Bronze Age industries^20,28–36^. These tracks have been important to understanding the ecology of *Homo sapiens* and its predator–prey interactions, and in determining the onset of cattle domestication. However, they were never formally described from an ichnotaxonomical point of view, and only rarely figured in discussions. At Formby, Sefton Coast (Liverpool) in England, tracks of aurochs, red deer, roe deer, and wild boar have been found in marshland deposits dated from 7.4 to 4.3 ka BP^37^. The co-existence of red deer, wild boar and aurochs seems to have been a common feature especially in the southern latitudes of Europe during the interglacial periods^38^. This is exemplified in Gibraltar, where the deposits of Genista I Cave provided remains of *Cervus elaphus*, *Bos primigenius* and *Sus scrofa* (reviewed in^39^). Coeval to this site is Solana del Zamborino, in Granada (Spain), where *Bos primigenius* was also found^40^. Dated as late Pleistocene (MIS 3), the painting of the Aurochs’ Hoofprint of Lascaux, with the clear representation of the dewclaws (digits II and V) in the posterior part of the track as being an important identifying feature when tracking aurochs^34,41^, is relevant to this study.

## Geological setting of the Trafalgar Cape

**Figure 2:**
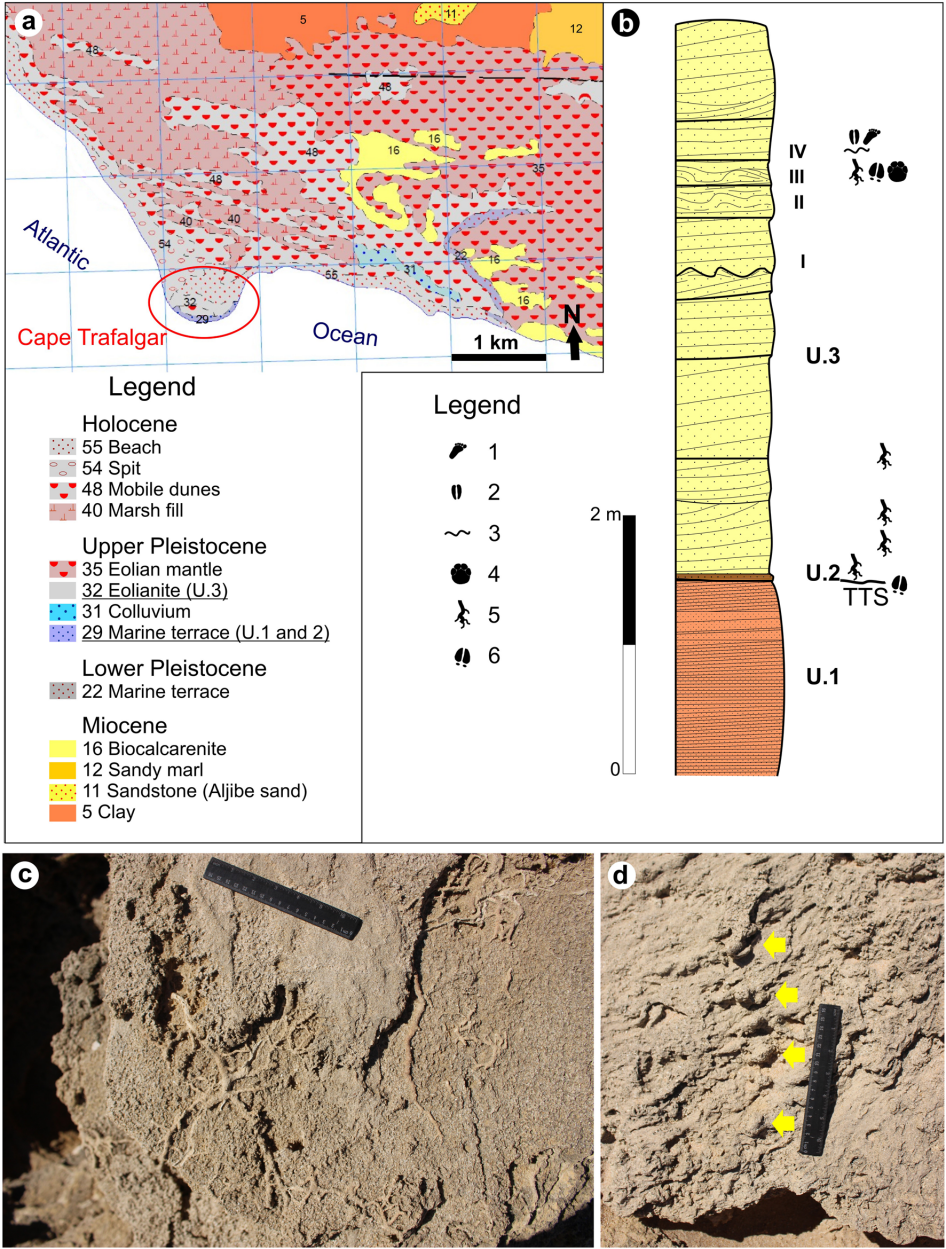
Stratigraphy of the TTS in Cape Trafalgar (Province of Cadiz). (**a**) Geological map of the Cap Trafalgar area (adapted from the Geological Map of Spain 1:50,000, Map no.1073 Vejer de la Frontera44, source: “© Geological Survey of Spain (IGME)”; (**b**) detailed stratigraphic column of Cape Trafalgar transition between beach and dune facies and location of the TTS and other trampled horizons (I–IV); rhizolith horizons corresponding to incipient paleosols are indicated (legend: 1—possible hominin footprint; 2—Pecoripeda isp.; 3—bioturbated lamina; 4—large footprint related to *Palaeoloxodon antiquus*; 5—rhizoliths; 6—*Bovinichnus uripeda*); (**c**) thin horizontal and densely branched rhizoliths from the second eolianite unit; (**d**) insect burrows in linear clusters preserved as convex hyporelief; ruler is 15 cm. Legend in (**a**), figure (**b**) and modifications to the photograph (**d**) have been made with CorelDraw 12 (https://www.coreldraw.com/la/).

The Pleistocene coastal deposits of SW Spain are distributed in short sections along the shores of the Gulf of Cadiz (Fig. 2). The Asperillo cliff in the Province of Huelva is one of the longest continuous outcrops of Late Pleistocene-to-Holocene deposits of the region, mostly composed of poorly-cemented eolianites and revealing at the base a paleosol developed over interdune pond deposits with a large trampled surface recently described as the Matalascañas Trampled Surface (MTS)^15,16^. About 100 km SE of the MTS is Cape Trafalgar, a tombolo connecting the 17 m-high paleodune, oriented WNW-ESE, with the mainland. The last interglacial-Holocene succession between Barbate and Cape Trafalgar rests unconformably on Upper Miocene deposits^42^ (Fig. 2a). The outcrop extends by a maximum area of 600 m by 300 m during low-tide, beneath the lighthouse. The succession (Fig. 2b) is composed of coarse-grained bioclastic sandstones, mostly massive but with a fine, almost horizontal lamination with a NE dip. This unit is truncated by an erosional discontinuity dipping south where abundant large artiodactyl tracks and rhizolith clusters can be found: the Trafalgar Trampled Surface or the TTS. Overlying the TTS and filling the tracks is a 40 cm-thick bed of very coarse, bioclastic sandstones with large broken bivalve shells and balanids, but also with occasional branched rhizoliths, mostly horizontal, up to 4 cm thick (Fig. 2c). The remaining succession is composed of eolianites made of medium-to-fine grained sandstones with planar cross-lamination. Reactivation surfaces are marked by the development of rhizolith horizons, and four (I-IV) highly bioturbated levels with large artiodactyl cloven-hoofed tracks were recognized in a 1.5 m- thick sequence composed of foresets dipping over 20º towards N (Fig. 2a). The succession is then partially covered by the Holocene dune system, but still shows around 2 m of eolianites with similar features to the ones mentioned below, including sparse bioturbation by isolated tracks, horizontal thin rhizoliths and insect burrows (Fig. 2d). This unit ends with a paleosol with a dense vertical rhizoturbation.

The basal beach unit from Cape Trafalgar was dated with U-Th from the last interglacial, MIS 5c (107 ± 2 ka)^42^. This sequence shows a disconformity where the TTS was developed, mostly representing the foreshore. The sand barrier retreat has exposed the TTS to the activity of large artiodactyls in the shoreline. The first eolianite unit was dated by OSL of 99 ± 12 ka^42^ and reveals the progradation of the dune system in a regressive context, including the development of rhizoliths downward to the disconformity and intersecting the tracks. The aggradation deposition of eolian dune sands is marked by high bioturbated levels and the formation of incipient soils with intense rhizoturbation. The second eolianite unit was deposited *circa* 78 ± 8 ka^42^.

When comparing this with the Asperillo cliff reference section in the Bay of Cadiz, according to the few OSL ages presently available^43^, the MTS was formed during the Last Interglacial warm and seasonal humid period (106 ± 19 ka) as a reddish paleosol (MIS 5), when the sea-level was at highstand position but with frequent fluctuations. Therefore, the TTS may be contemporary of the MTS, and both highlight the presence in the shore of large artiodactyls (see below).

## Systematic ichnology

Nomenclatural acts: This study follows the requirements of the International Code of Zoological Nomenclature regarding the registration of the nomenclatural act in ZooBank.

Ichnogenus: *Bovinichnus* igen. nov.

Type ichnospecies: *Bovinichnus uripeda* isp. nov.

Etymology: referring to traces (= *ichnus*) with the morphology of tracks shared by the species of the Tribe Bovini, subfamily Bovinae, which includes besides *Bos*, buffalos, bisons, among others.

Diagnosis: Tetradactyl, paraxonic and subsymmetrical tracks that are as wide as long; central (III–IV) digit impressions are subparallel, with broad distal end pointing forward, and a narrow, linear interdigital space; internal surfaces of hoofprints flat to slightly concave, external surfaces slightly to markedly convex; hoofprints widest near the heel, but tapering only to minor degree between the heel and the apex; rounded-to-rectangular posterior dewclaw (digits II and V) prints that are much smaller than central digits, printed next to or close to the heel; *manus* and *pes* of closely similar form and size.

*Bovinichnus uripeda* ichnosp. nov.

ZooBank—urn:lsid:zoobank.org:pub:E834D014-3A20-4721-9B2D-4577B2E10A52.

Koenigswald et al., 1996: Fig. 3E.

Roberts, 2009: Figs. 13, 14.

Neto de Carvalho et al., 2020: Fig. 2A,C,D,E.

Holotype and Paratypes: TTS10 (Fig. 5a), and TTS8, TTS13, respectively. They were left in situ but photogrammetric 3D models were produced (Fig. 4).

Type horizon: Trafalgar Trampled Surface (TTS): Fig. 5a–c.

Etymology: In Latin, the prefix *uri*- is the plural word for aurochs and the suffix -*peda* refers to feet.

Type locality: Cape Trafalgar, GPS: 36°10′54.92′′N–6°02′05.87′′W.

Diagnosis: The same as for the ichnogenus.

**Figure 3:**
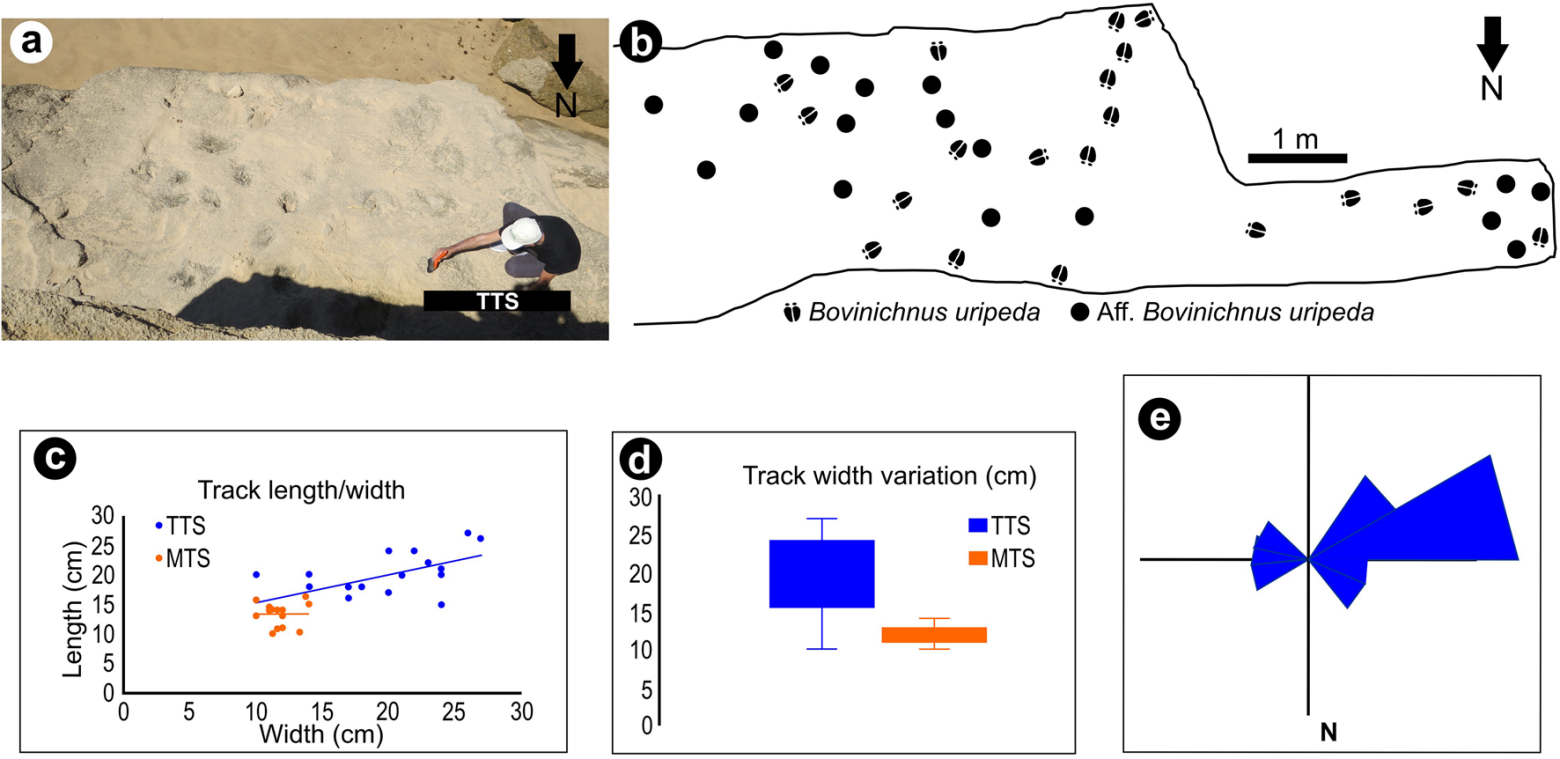
Graphical representation and data on measurements taken in the TTS and the MTS: (**a,b**) Distribution of tracks in the main area of the TTS; (**c**) bivariate plot of track length/track width ratio (n = 18 in the TTS and n = 17 in the MTS). The track length included dew claw impressions if present; (**d**) boxplot of track width variation (n = 18 for the TTS and n = 17 for the MTS); (**e**) rose diagram with the orientation of the hoofprints in the TTS (n = 14). Figure (**b**) has been drawn with CorelDraw 12. (https://www.coreldraw.com/la/).

**Figure 4:**
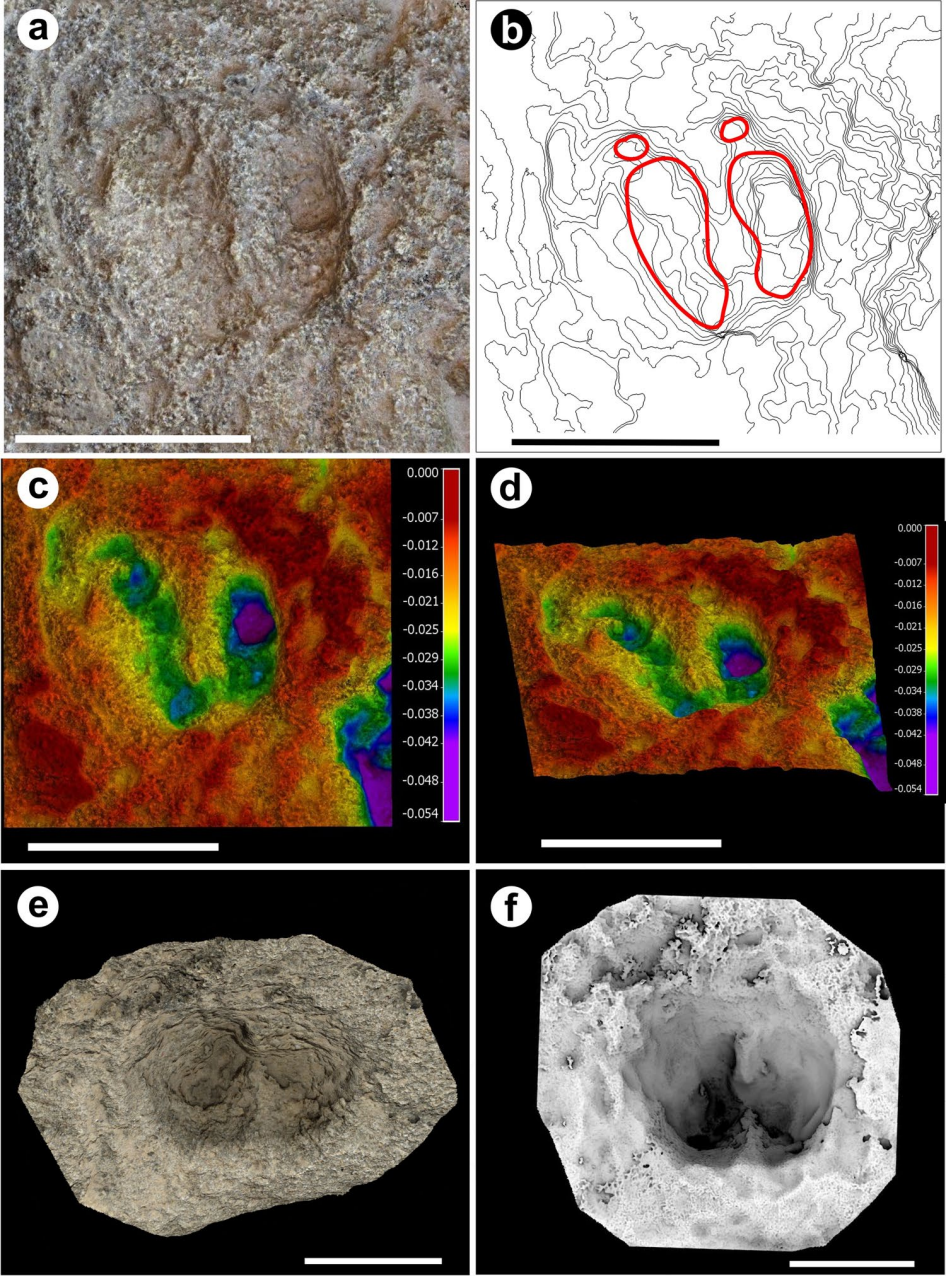
3D models of the *Bovinichnus uripeda* holotype (TTS10) and paratype TTS8: (**a**) Orthogonal view of the natural colours textured 3D model of the TTS10 with the general morphology depicted from the contour lines map, equidistance of 1 mm (**b**); (**c**) false colour DSM in orthogonal view showing deeper areas in the heel side of the hooves and the tips resulting from the impulse created in the foot-off event. The circular-to-elliptical dew claw imprints are also evident behind the hooves; (**d**) oblique view of the previous DSM to complement previous observations; (**e**) oblique view of the textured natural colour 3D model of the paratype TTS 8; (**f**) orthogonal view of the paratype TTS 8 where the deepest part of the hoofprints is displaced to their anterior part reflecting the pressure angle exerted by the limb on the substrate during the foot-off event. Scale bar is 150 mm. The 3D model of the images (**a–d**) were produced by the software WebODM 2.1.0, https://github.com/OpenDroneMap/WebODM; MeshLab 2020.12, (https://www.meshlab.net/); CloudCompare 2.11.0, (https://www.danielgm.net/cc/) and the 3D model of the images (**e,f**) were produced with the software Meshroom 2021.1.0, (https://alicevision.org/#meshroom); MeshLab 2020.12, (https://www.meshlab.net/); CloudCompare 2.11.0, (https://www.danielgm.net/cc/).

**Figure 5:**
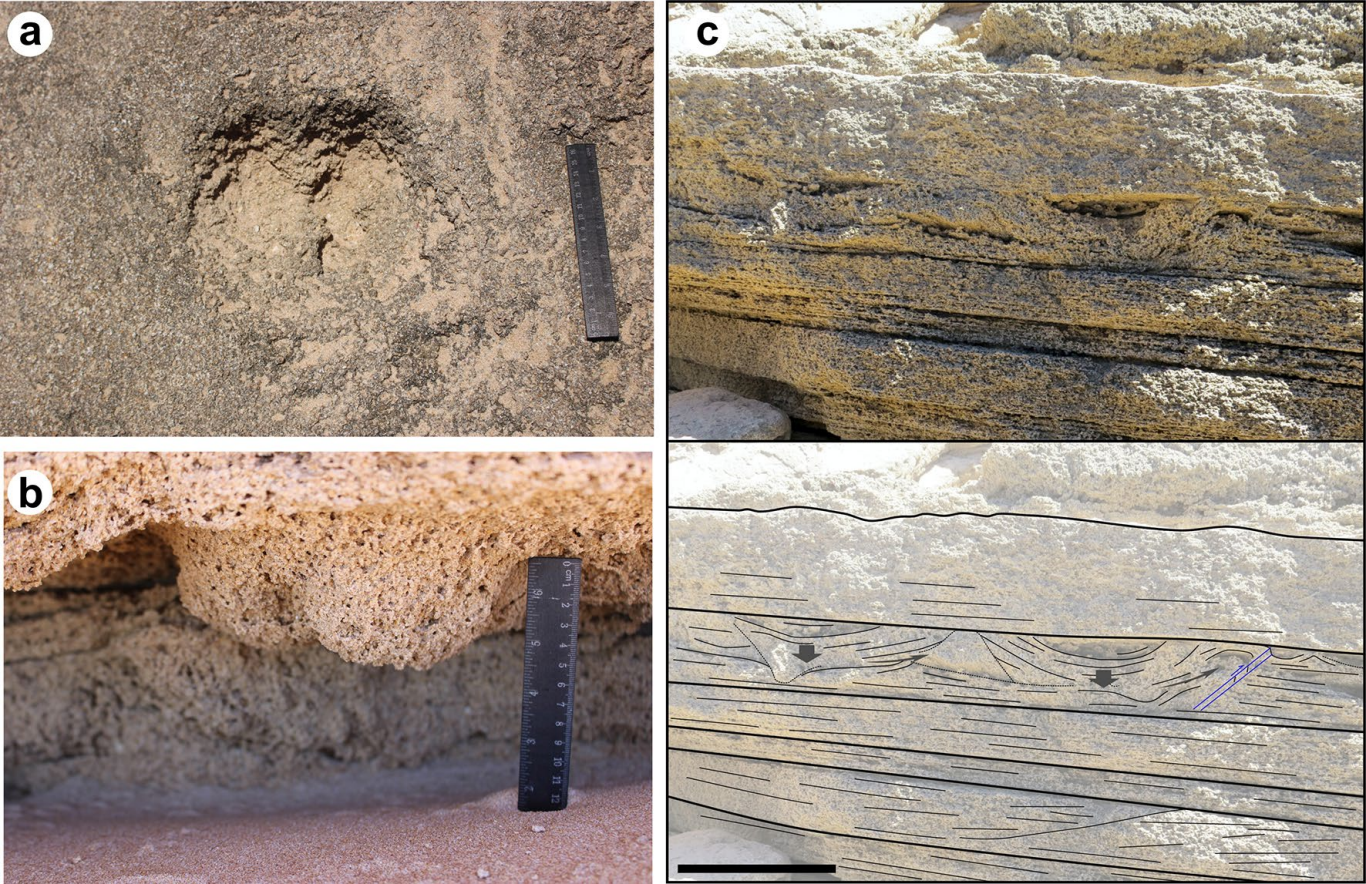
*Bovinichnus uripeda* igen. nov. et isp nov. from the Late Pleistocene (MIS 5c) of Cape Trafalgar: (**a**) well-preserved track as concave epirelief (holotype TTS10) showing the dewclaws; ruler is 15 cm; (**b**) convex hyporelief preservation in the trampled horizon I, evidencing the narrow ridge between toes; (**c**) tramped level IV with large bifid tracks attributed to aurochs in section (above) and interpretation of the sedimentary deformation (below). Scale bar is 15 cm. The interpretation of (**c**) (below) was drawn with CorelDraw X7 (https://www.coreldraw.com/la/).

Description: Tracks occur in the TTS in concave epirelief, mostly isolated, or in small *manus-pes* sets up to seven in the same trackway (T3) (Fig. 3a,b). The breadth size range varies between 100 and 270 mm, but with most of the tracks measuring 200–240 mm (Fig. 3c,d). They are mostly oriented towards WSW (Fig. 3e) determined by the tipping of the hoofprints or the location of the dewclaws (Fig. 4a,b). Usually, well-preserved true tracks are tetradactyl and show evidence of pressure pads. The digits III and IV are by far the largest and correspond to crescent-shaped impressions. The posterior part of the prints is deeper than the cleave or digit tips and may reveal rounded-to-rectangular small dew claw imprints proximal to the heel. The outer width measured between dew claw impressions corresponds in size to the posterior width of the hoofprints (Fig. 4). The termination of each hoof impression is blunt and directed forward. In convex hyporelief preservation and cross sections, hoof imprints are separated by a central ridge (Fig. 5b). At least four trampled levels can be seen in cross section in the eolianite beds. The indentation produced by the large rigid hoof over the well-laminated sandstone show soft deformation, composed of marginal upfolds and microfaults, of the underlying laminae and a concave track infill (Fig. 5c,d). The stride length, or distance between footprints from the same foot, calculated for the trackway T3 varies from 210 to 100 cm and is 150 cm in the trackway T1. The pace length, or the distance between two successive footprints, varies from 110 to 45 cm in T3 and is 96 cm in the trackway T2. There is no evidence in trackways for *manus-pes* overprinting and 'direct register', in which pes is placed directly in manus impression, or in the cross sections of the trampled surfaces in the eolianite.

Tetradactyl tracks with the morphology of *Bovinichnus uripeda* were previously described in the MTS^16^. They are now synonymized under *B. uripeda* despite showing smaller dimensions (Fig. 3c,d).

**Figure 6:**
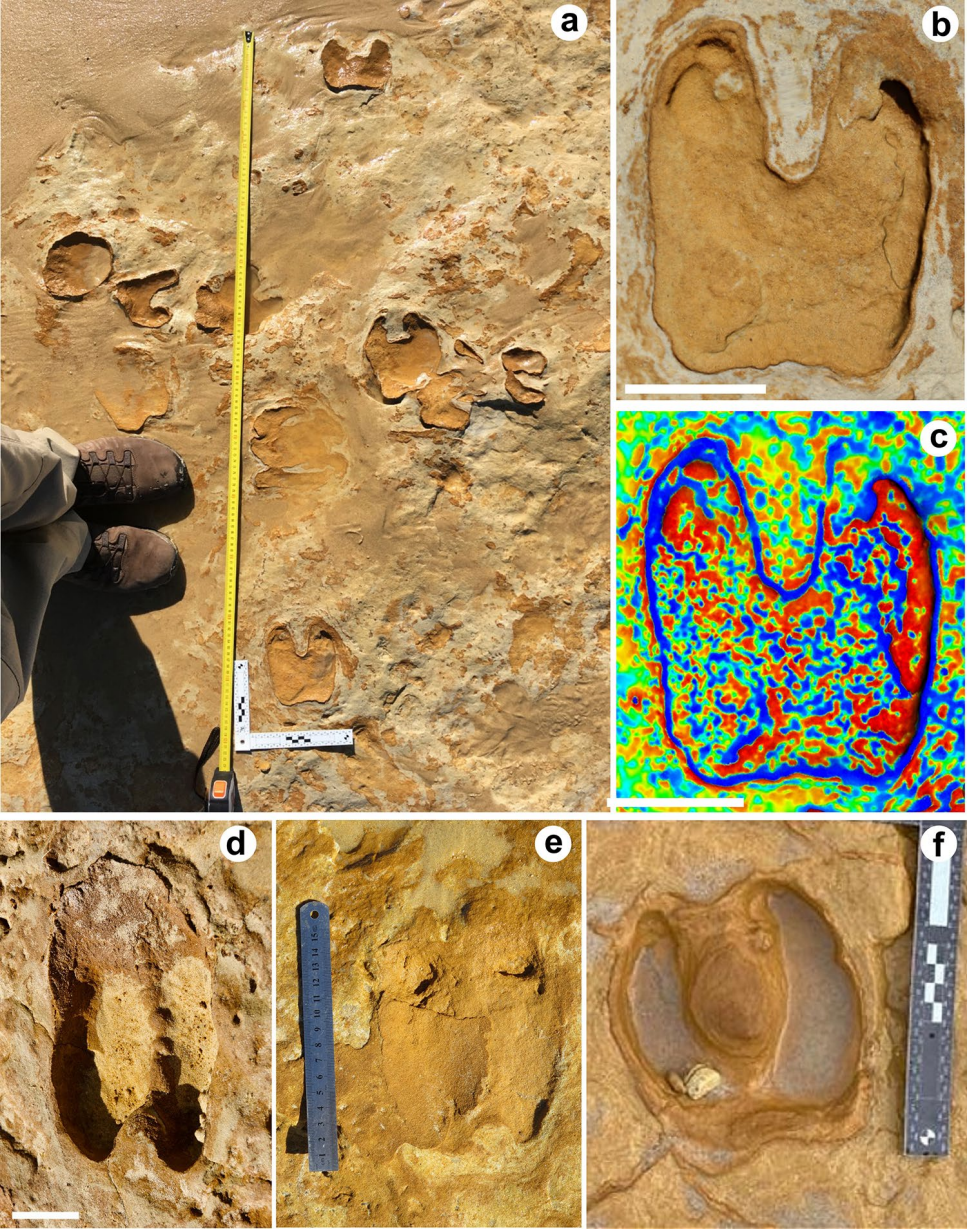
Large artiodactyl tracks from the MTS. (**a**) Trackway T1 attributed to aurochs based on the large size of the squared tracks and the preservation of the dewclaws as rectangular imprints; (**b**) 3D model of a tetradactyl track from the MTS interpreted as *Bovinichnus uripeda*, vertical view of the texturized 3D model in natural colours; (**c**) vertical view from the previous track made of the false colours DSM (cold and hot colours represent depth gradient, from deeper to shallower, respectively); (**d**) *Bovinichnus uripeda*, large track; (**e**) *manus-pes* couple, note the dewclaws; (**f**) large cervid track found in the MTS for comparison, with slender toes with sharp tips, well splayed, with almost linear outlines. Scale bar is 10 cm. The 3D model of the images (**b,c**) were produced by the software Meshroom 2021.1.0 (https://alicevision.org/#meshroom); MeshLab 2020.12 (https://www.meshlab.net/); CloudCompare 2.11.0 (https://www.danielgm.net/cc/).

The morphotype A1 described in the MTS as *Cervipeda*^15^ corresponds to large hoofprints with an average size range of 12–13, 5 cm in length and 7.5 cm broad and some few trackways with measured stride lengths between 100 and 132 cm. However, some tracks are up to 17.8 cm in length and show pace lengths of 100 cm, which are now integrated in the new *Bovinichnus uripeda* (Figs. 3c,d and 6a). The tracks can be rectangular or squared to oval in outline. Impressions of third and fourth toes are central, well-developed mirror images with internal surfaces sometimes in median or posterior contact, strait or concave at the front (Fig. 6b,c); in some examples, they are separated by a continuous interdigital space, usually broadest at the front (Fig. 6d,e). They can be widest near the heel or in the central part, tapering to an apex of angular or sharply parabolic shape, directed forward, or rotated inwards or outwards, to the midline of the trackway. The second and fifth toe impressions (dew claw imprints) can be seen in some tracks, are smaller and subtriangular or rectangular, located immediately behind the hoofprints (Fig. 6e) or separated from them, extending the tracks for extra 30 mm (Fig. 6c). The trackways show no major differences in size between fore feet and hind feet (Fig. 6a). Pressure pads surrounding the track indicate they were imprinted directly on the surface.

When not showing dew claw prints, the Matalascañas hoofprints are similar to ichnogenus *Bifidipes* Demathieu et al. They are didactyl footprints organized in homopod trackways with cleaves pointed and hooves diverging and clearly separated, although not so much closer, and wider, to the heel^45^ (Fig. 6e).

**Figure 7:**
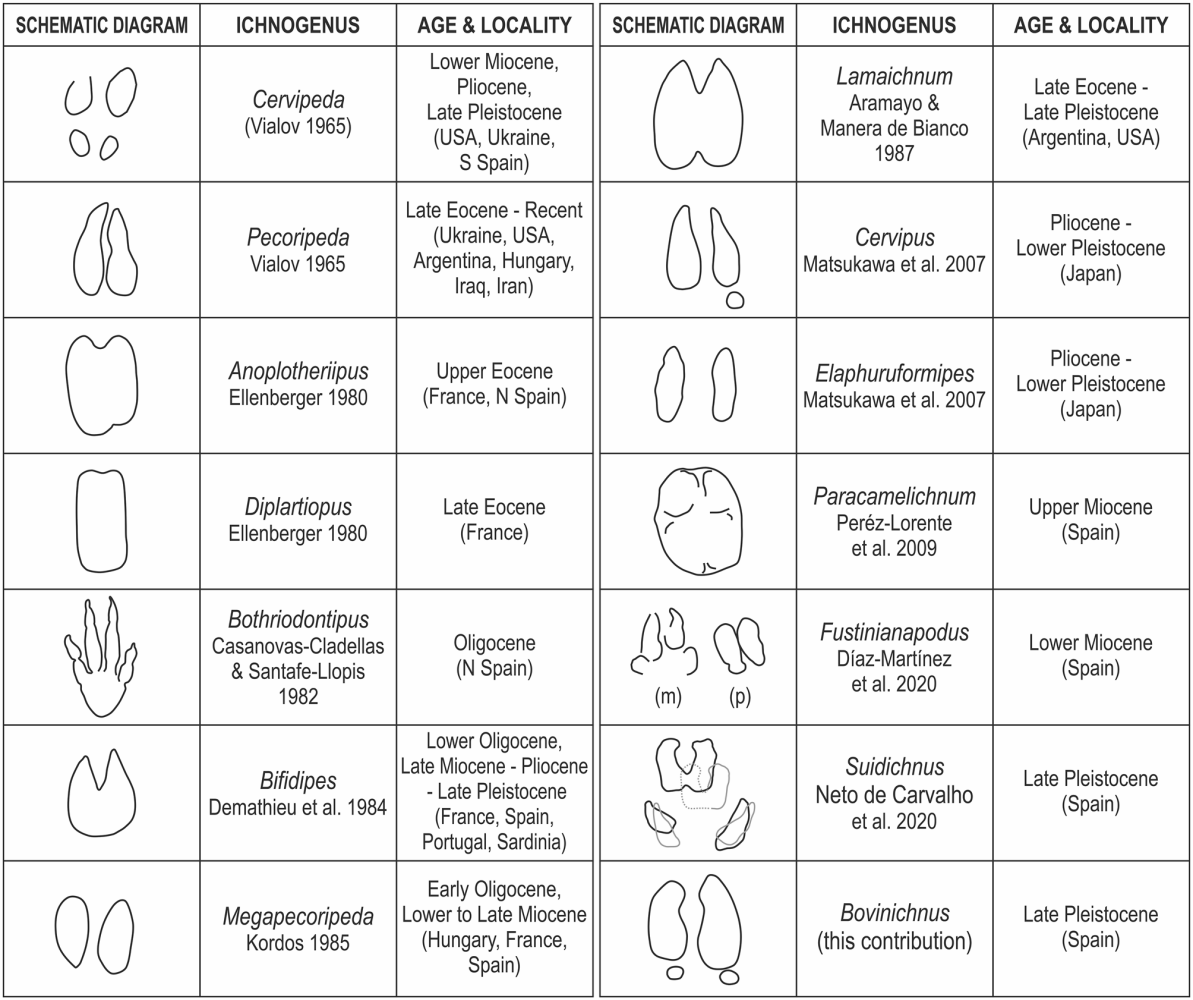
Revision of the valid artiodactyl ichnogenera^16^ with visual comparison of morphologies, age and locality (country). All tracks have been redrawn from the original references where they were described for the first time. (m) *manus*, (p) *pes. Anoplotheriipus* left *pes*; *Diplartiopus* left *pes*; *Bothriodontipus* pes (not indicated which side); *Lamaichnum manus* (not indicated which side); *Paracamelichnum* right *manus*; *Fustinianapodus* left *manus* and *pes*; *Suidichnus* overprinting of left *manus* by corresponding *pes*; *Bovinichnus* right track; for other ichnogenera autopods were not specified/shown no morphological distinction in the references. This figure has been drawn with CorelDraw X6 (https://www.coreldraw.com/la/).

Remarks: Sarjeant and Langston^6^ provide an early revision of artiodactyl fossil tracks emphasizing the lack of ichnological description of kidney-shape to crescentic hoofprints of many bovoids. The ichnotaxonomy of artiodactyl tracks is problematic since the hooves are characterised by a wide range of small morphological variations^6,22^. In this regard, there were attempts to revise the ichnotaxonomy of the artiodactyl tracks^6^ with the last made by Neto de Carvalho et al.^16^, and completed in the present work (Fig. 7). In this study the number of digit impressions in the well-preserved true tracks has been considered as the most important ichnotaxobase, thus *Bovinichnus* tracks were compared only with artiodactyl tetradactyl ichnotaxa that are presently valid^22^. Tetradactyl tracks attributed to artiodactyls are resumed to *Cervipeda* (Vialov), *Bothriodontipus* Casanovas-Cladellas and Santafé-Llopis, *Cervipus* Matsukawa et al., *Fustinianapodus* Díaz-Martínez et al., and *Suidichnus* Neto de Carvalho et al.. *Bothriodontipus* has four forward-direct ungual prints while *Bovinichnus* shows two large hoofprints and two posteriorly-located dewclaws. Both *Cervipeda* and *Cervipus* shows slender hoofprints and their largest size is in average much smaller than *Bovinichnus uripeda*. *Fustinianapodus* described recently in the Lower Miocene of Spain^22^ is recognized by subtriangular dew claw impressions in the *pes* tracks, and elongate ones in the *manus* tracks. Finally, *Suidichnus* described in the MTS are tracks longer than wide, with a general trapezoidal shape, and dew claw impressions conical to comma-shaped, smaller than the main toes, longer than wide, and projected laterally from behind the main toes, with a wider angle in the foreprint comparing with the hindprint^16^. Artiodactyl tracks with hooves parallel but with hoof apices almost as rounded as the hind portions, the track having an oval to rounded-rectangular outline, are placed into *Lamaichnium* Aramayo and Bianco^6^. Besides that, the didactyl shape of these tracks attributed to camelids clearly distinguishes it from *Bovinichnus*. Therefore, and following the suggestion of Sarjeant and Langston^6^, *Bovinichnus uripeda* is considered a new artiodactyl ichnotaxon of bovoid morphology.

Slow walking usually keeps *manus* and *pes* separated in the artiodactyls^46^, with *manus* impression lying ahead of the *pes* impression. Stride over 2 m together with the shape of cleaves and the large width of the prints indicate that the TTS were produced by large bovines. In the Sefton Coast and at Peterstone (England), aurochs tracks associated with Chalcolithic industries, show lengths of 220–280 mm^37^, which are also comparable in shape with *Bovinichnus uripeda* and must be included in the new ichnospecies (see also^34^). Finally, the Late Paleolithic rock art painting of the Aurochs’ Hoofprint of Lascaux (France), with the clear representation of the dewclaws in the posterior part of the track, recognizes the tetradactyl shape of aurochs tracks^34,41^.

The tetradactyl A1 morphotype described in the MTS shows some differences that allow to separate into two different morphotypes (Fig. 6). Despite the preservational variation in morphology that can be found in the MTS^15^, and the fact that in average A1 tracks have the same size, there are subtle differences that suggest different producers. The larger tracks, blunt shape of cleaves without separation in the proximal area and their convex outline, making many of them rounded or squared, with circular to oval dew claw imprints, can be compared with *Bovinichnus uripeda* from Cape Trafalgar and the tracks of the aurochs known in the Holocene deposits of Great Britain^34,47^ (Fig. 6a–e). On the other hand, cervid prints are slenderer with the previous half of each foot tapering to a point, for example, in red deer (*Cervus elaphus*) the forefoot generally creates splayed tracks, especially in soft and/or sandy grounds (Fig. 6f).

## Discussion

### The difference between red deer, domestic cattle and aurochs tracks

Despite red deer, domestic cattle and aurochs tracks may show a tetradactyl preservation, red deer tracks are slenderer, with sharp cleave anteriorly, and cattle leave rounded tracks with convex outlines, when compared to aurochs. Large artiodactyl hoofprints attributed to cervids have been described in the Late Pleistocene of the Iberian Peninsula, including Gorliz in northern Spain, SW Portugal and the MTS^10,13–15^. These longer-than-wide tracks composed of hoofprints that are more slender than those of *Bovinichnus uripeda*, can reach up to 150 mm in length; the tracks from Gorliz attributed by Flor^10^ to *Cervus elaphus* (see its Fig. 5) reach 130 mm in length but are rounded and could have been produced by the reindeer (with the widest and roundest tracks of all deers) or the European Bison. In the Sefton Coast, the red deer tracks dated from the Neolithic are 100–150 mm in length^37^, while modern average of a full-grown red deer stag’s fore print is 80–90 mm long and 60–70 mm wide^46^. Pointed cleaves of the red deer contrast in this area to the more rounded and larger hooves of the aurochs. Also, the outer edge of the cleaves curve evenly towards the tip. In red deer, the dewclaws are rounded impressions. Straddle is relatively narrow, with a stride length for adult varying between 80 and 150 cm^46^.

Barr and Bell^20^ interpreted rectangular hoof prints, ranging from 120 to 180 mm in length, as made by red deer and the larger square hoof prints, ranging from 200 to 260 mm in length, as made by aurochs. When showing similar sizes, aurochs differ from cervid tracks by the blunt tips and wider hooves, besides the smaller space enclosed between the two hooves in aurochs^48^.

Despite being broadly bovoid rounded tracks, there is a marked difference in size, especially in length, between *Bos taurus* and *Bos primigenius*. Tracks of extant, fully grown cattle are ~ 100 × 95 mm in size^20^, which is less than half the size of the very large bovine tracks found in Cape Trafalgar. For the purpose of comparing the morphology (but not taphonomic variation) of extant domestic cattle hoofprints with Cape Trafalgar and Matalascañas tracks, a bull 1128 kg in weight was invited to walk in a dry fine sand substrate. He left rounded tracks measuring 180 mm long which are nevertheless more rounded than aurochs tracks. Moreover, the hooves outside are convex and the inner edge is concave at the front and convex at the back^46^.

### Aurochs vs. Bisons as producers of *Bovinichnus* in SW Iberia

Aurochs and Steppe Bisons coexisted in Iberia during the Late Pleistocene. Since these two bovines were in size and morphologically very similar, these would rise the possibility that steppe bisons could be likely producers of *Bovinichnus*. Therefore, it is vital to understand the biogeographical distribution of the two species in order to be sure about the most likely producer of *Bovinichnus uripeda* in SW Spain.

The origin of the aurochs is not clear, although it is thought to have Indian ancestry, dating back to about 2 Ma^49^. The aurochs were first reported in Europe during the Middle Pleistocene at Venosa-Notarchirico, Italy, and finally became extinct in Poland in 1627 AD^38^. Aurochs finds are less numerous during the Pleistocene than during the Holocene, but a large distribution area is represented nonetheless, from SW Iberia to southern Scandinavia, North Africa to Korean peninsula and Japan^50^. In the Eemian (MIS 5e), they expanded towards northern territories, where their skeletons were found, e.g., in excavations made in the Trafalgar Square at central London^51^. During the early Holocene (Mesolithic and Neolithic cultural phases in Europe) the species seems to have increased in number, probably due to increasingly favorable mild and wet conditions after the end of the Last Ice Age^50^. Its early appearance in Spain dates back to 0.7 Ma^52^. Remains of aurochs dated from the Middle or Late Pleistocene have been uncovered in Great Britain, France, Spain, Portugal, Italy, and Germany. The distribution of the aurochs has fluctuated with the changing climate during the Pleistocene, being more widely dispersed in interglacials and interstadials^50^. During the Pleniglacial, with the European northern ice cap boundary having a southerly course, and with the Iberian and Italian peninsulas being used as refugial areas^53^, aurochs became rare or completely disappeared during colder periods in many areas of Europe. At Gibraltar, taking advantage of the milder climate in the southernmost Europe during the MIS 3, *Cervus elaphus*, *Sus scrofa* and *Bos primigenius* were found related to habitats of stone pine/juniper woodland, savannah, shrubland with patches of grassland^54^.

The long-horned *Bison priscus* (Bojanus), or steppe bison, is the earlier form of *Bison*, thought to have become extinct in Europe at the end of the Pleistocene^55^. As the aurochs, the steppe bison had also a wide distribution across Europe, from the Iberian Peninsula, through central and eastern Europe and into Siberia, crossing the Bering strait into North America during the early Holocene^56,57^. The steppe bison was able to adapt to a wide range of environments, both glacial and temperate^53^, by performing long migrations. Of the two species, *Bison priscus* is thought to be particularly problematic in terms of its skeletal distinction from *Bos primigenius*^58,59^. The problem is enhanced by the fact that *Bison priscus* was relatively abundant during the Pleistocene, and seems to appear alongside *Bos primigenius* on several sites. Nevertheless, the steppe bison is known only from MIS 3–2 from northern and central Spain^18,26,60^ during which it was found together with *B. primigenius*^25^. During this period, the expanding territories of *B. priscus* related to colder and steppe environments may have reached the southern latitudes of Granada^61^. *B. priscus* appears in the cave art from North (Altamira, Altxerri, Santimamiñe and Ekain) and Central Spain (La Hoz and Siega Verde) during the Last Glacial Maximum, where representations of aurochs are rare^18^. Bison are absent from the long and well-studied Late Pleistocene sequences in Gibraltar, and elsewhere in SW Iberia. Thus, the aurochs were the only bovines existing during the MIS 5 in SW Iberia and we can confidently attribute the *Bovinichnus uripeda* tracks from Cape Trafalgar and Matalascañas to aurochs.

The only fossil tracks related to *Bison *sensu lato that are known were described^62^ in dune and interdune deposits from Nebraska (USA). However, they are found mainly in cross sections, as concave-up deformation structures or undertracks, circular to oval in plane, ranging from 70 to 160 mm in diameter. Due to this kind of preservation, it is not possible to compare the Nebraska tracks with *Bovinichnus uripeda*. However, if we compare the tracks produced by the extant *Bison bonasus* with *Bovinichnus uripeda* we realize they are wider than long and, therefore, do not present the more rectangular morphology (slightly longer than wide) evidenced in *Bovinichnus*. Therefore, the track length/width ratio can be very useful to distinguish between the presence of aurochs and steppe bisons in the track fossil record.

### “The Beach Bulls”: large aurochs tracks in the TTS

**Figure 8:**
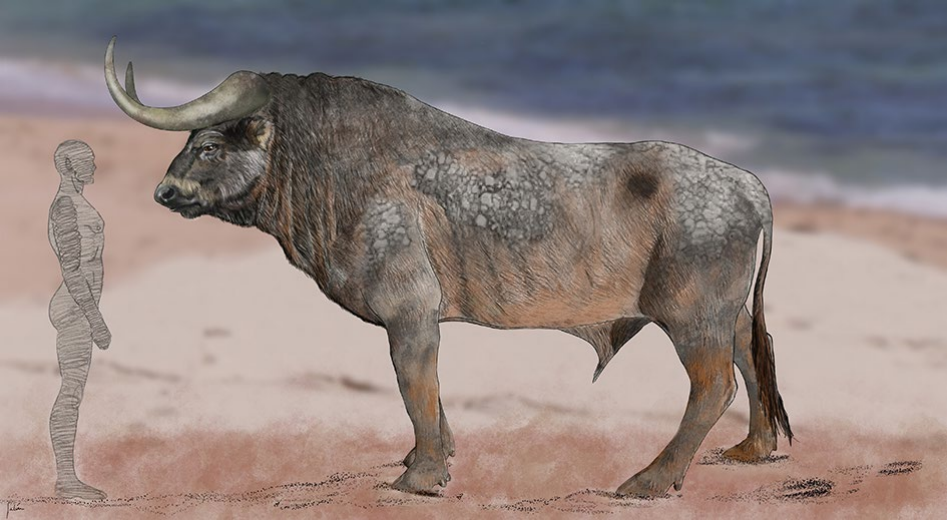
Reconstitution of the size of the aurochs based on the maximum size of the tracks found in the TTS, in a context of beach environment where they were produced (artwork of José María Galán).

Aurochs have reached their maximum body size in the early Late Pleistocene interglacial^38,61^. There is an increase in size of the metapodial bones preserved in levels dated as Middle Pleistocene to early Late Pleistocene and a decrease of the same during the Late Pleistocene to early Holocene^38^. This trend is also found for other large mammals in Europe such as the red deer^63^ and the wild boar^64^ (see evidence from tracks in^16^). After that, there is a general size decrease between the Late Pleistocene and the early Holocene^52,58,64,65^, with a size increase recorded after beginning of the Holocene^66^. The Last Glacial was responsible for a decrease in size of the aurochs^47,67^. There is also clear evidence of a size reduction in red deer at the end of the Pleistocene in Iberia^67,68^. In the case of the aurochs, the body size increase during the early Late Pleistocene may be related to predation pressure since during this period these animals were prey for a large array of large carnivores, including Neanderthals. The large track size recorded in the early Late Pleistocene from Cape Trafalgar (Fig. 8) is comparable with the records of up to 280 mm in length from the lower Holocene of western England^37,47^, which correspond to the largest track record attributed to aurochs. This increase in size of aurochs recorded in tracks during the Holocene has been attributed to predation pressure and husbandry^20,69^.

### Aurochs visiting the beach

Aurochs was typically a “wetland specialist” which preferred to live in marshy forests, near estuarine areas and floodplains for grazing during the day^50,69^. According to these authors, aurochs lived in small herds and were grazers, not browsers like many deer species, thus requiring open landscapes composed of grasslands. For this main reason, herds of aurochs were roaming the coastal areas of southern Europe during the Late Pleistocene. Pandolfi et al.^38^ have described remains of aurochs together with red deer and wild boar in southern Italian coast referable to early Late Pleistocene. In Portugal, *Bos primigenius* osteological remains are found in the Atlantic coastal caves of Figueira Brava (MIS 5), Furninha (MIS 4–2?), Lapa da Rainha (MIS 2), and Algar de Cascais, besides close to the alluvial plains of Tejo River, at Foz do Enxarrique and Salemas quarry (MIS 3), and further south, Vale de Boi and Lapa do Picareiro sites (MIS 3–2)^17,70–72^. As described before, aurochs were also found in caves of Gibraltar, not far from Cape Trafalgar, in contemporary and later Pleistocene deposits (MIS 5–3)^39,54^. In the Mediterranean coast of Málaga, Zafarraya (MIS 3) and Nerja (MIS 2) caves show the already rare presence of aurochs, besides being pictured in the rock art from Navarro (MIS 2) coastal cave^73,74^. Finally, the Valencian coast shows the long presence of aurochs between MIS 9–2 in the caves of Bolomor, Cova Negra, Beneito, El Salt, and Abric del Pastor^75^. The aurochs tracks from early Late Pleistocene of the TTS and the MTS add two more coastal sites to the ones listed in southern Iberia.

The aurochs track record and skeletal remains are abundant in Late Pleistocene to Holocene estuarine paleoenvironments in Great Britain^20,34,47^ where they were preserved in intertidal deposits^30,70^. Glades may have attracted the animals to graze not far from the dense coastal woodland^37,70^. The TTS and the MTS are located not far from the marshlands of Cadiz, and the Guadalquivir and Odiel rivers, respectively. But their association to beach and dune-interdune deposits, not in direct relation with fluvial environments, requires further explanation. This explanation may be found in a modern analogue example from the Doñana National Park, by comparing the behaviour of wild cattle (*Bos taurus*) regarding resources partitioning between seasons.

Doñana feral cattle has been living under free-ranging conditions since the thirteenth century^76^. High quality habitats in Doñana National Park include lagoon meadows and the lower grassland of the ecotone zone between the scrubland and the marshland, which are most used by the active habitat selection of the feral cow-herds^76^. Land sand dunes found in large areas in the coast of Doñana (primary or covered by a xerophytic community of shrubs) are considered a low-quality habitat where the availability of the forage is low and therefore avoided by the cattle. Most of the tracks in the MTS likely attributed to aurochs are smaller in size than *Bovinichnus uripeda* described in the TTS. This track size difference in coeval deposits may be explained by habitat difference/preference and the strong sexual dimorphism known for aurochs. Lagoon meadows related with interdune drying ponds was the most likely environment described in the MTS^15,16^. Aurochs, especially cow-herds would have centred their movements around food patches of high availability and quality of food, such as the MTS. This could happen especially at the end of spring and during summer, providing enough food for the offspring. However, in the coastal beach and dunes of Trafalgar Cape the aurochs presence recorded by larger-sized tracks and trackways, in the TTS showing a preferential orientation of movement towards the shoreline. The well-drained sandy substrate certainly resulted in the virtual absence of an herbaceous layer in this beach area, where also the eolianites show a sparse rhizoturbation.

Among dimorphic mammals, males have higher metabolic needs than females and are usually relegated to low quality habitats, resulting in male greater seasonal home range^76^. The tracks in the TTS are mostly from large bulls and may indicate this wide dispersion to poor-quality coastal habitats. But there are few tracks 100 mm in length that can be related with the presence of cows. In Doñana National Park, this occasional presence of both cows and bulls together in coastal habitats usually happens in autumn, when rut is in its peak and the bulls were associated with the cow-herds^76^. Nevertheless, cow-size tracks in the TTS are rare comparing with the large tracks and trackways, showing mostly the same orientation, which can only be attributed to bulls walking together in herd. Therefore, while the MTS may record the presence of a cow-herd, the five trampled horizons in Cape Trafalgar may have resulted from the occasional but recurrent procurement of the shore by small herds of bulls.

It is relatively common nowadays to find feral cattle visiting the shores across the world. The Xhosa beach cattle, the “beach-loving cows” in South Africa, even became a tourist attraction. They come down to the shores usually to curl up on the sand and chew the cud. Other examples of recurrent visits of herds to the shores across the world may be found in Hong Kong or Corsica. The Cape Trafalgar’s TTS might represent the arrival of a “beach-loving” aurochs herd of bulls for enjoying digestion while controlling possible predators in open landscape, or have a short moment of rest from parasites.

## Conclusions

The dated early Late Pleistocene (MIS 5) coastal deposits from SW Iberia show the presence of artiodactyl tracksites. The new Cape Trafalgar site (TTS) together with the recently described Matalascañas site (MTS) record the recurrent trampling by large cloven-hoofed ungulates, being the oldest record of aurochs locomotion. *Bovichnus uripeda* igen. *et* isp. nov. is formally described and safely attributed to aurochs, after size and morphological comparisons with coeval large red deer tracks and the biogeographical distribution of the steppe bison, only present in southern Spain during the MIS 3. Other artiodactyl tracks, together with elephant and human tracks (see Supplementary Material I) help to provide a perspective of the aurochs ecological community, both in the TTS and the MTS. The unexpected paleoenvironments where the trampled horizons occur, i.e., beach and dune deposits, in the TTS, and interdune pond deposit, in the MTS, suggest a resource partitioning comparable with the cow-herds vs. bull-herds found nowadays in close related feral cattle (*Bos taurus*) living in the nearby Doñana National Park. Smaller tracks in average found in the MTS may represent cow-herds grazing in a high-quality food patch related with the retreating seasonal pond and development of meadows. The large tracks from the TTS described as *B. uripeda* and showing mostly the same direction of movement, are better interpreted as a bull-herd slowly walking towards the shoreline and looking for some peace, as it presently happens with feral cattle visiting the shores in different parts of the world.

## Materials and methods

Field campaigns in the Cape Trafalgar section, made between 2020 and 2021, produced 2D cartography, 3D photogrammetric models of the most representative tracks and a detailed stratigraphic log. This data was then compared qualitatively with the large artiodactyl tracks that have been described in the MTS^15,16^.

### The ichnological material

The Cape Trafalgar section provides at least five stratigraphic horizons with large cloven-hoofed tracks. The upper four horizons can be seen mostly in section as undertracks and preserved as true tracks in bedding soles. There, it was not possible to identify trackways. The TTS is exposed for an area of at least 70 m^2^ and 36 tracks were positively identified. Tracks are concentrated in the eastern sector of the TTS, but at least 9 tracks can be found towards west, with at least two of them belonging to the same trackway (T1). Six short trackways, composed of two to seven tracks sets, were determined in total. Each track was provided with a code (TTSx) and measured (Table 1). Well-preserved hoofprints were measured regarding length, the breadth of the widest part of the print, length and breadth of the dewclaws whenever present, orientation, pace and stride when organized in trackways. Track length/width ratio was calculated and may be used to distinguish artiodactyl tracks in the fossil record with comparable morphologies, e.g., *Bison*. Track depth was indicated only for the deep tracks since most of the tracks show no more than 20–30 mm in depth. However, several tracks are just rounded prints without evidence of toes. In these cases, only rarely measurements were obtained using the pressure pads to orientate the track.

**Table 1:** Track measurements in the TTS and the MTS.

Reference	Length (cm)	Width (cm)	L/W ratio	Depth (cm)	Shape	Orientation	Trackway
TTS1	14	10	1.4	–	Hoofprint	230°SW	–
TTS2	10	20	0.5	–	Hoofprint	80°E	–
TTS3	20	24	0.83	9	Hoofprint	275°W	T2
TTS4	17	16	1.06	14	Hoofprint	303°NW	
TTS5	17	18	0.94	–	Hoofprint	109°E	
TTS6	27	26	1.04	–	Hoofprint	259°W	T3
TTS7	21	20	1.05	–	Hoofprint	259°W	T3
TTS8	20	17	1.18	2.3	Hoofprint	259°W	T3
TTS9	24	20	1.2	–	Hoofprint	122°SE	–
TTS10	22	24	0.92	2.7	Hoofprint	250°W	T4
TTS11	24	15	1.6	–	Circular	–	T4
TTS12	23	22	1.05	–	Hoofprint	250°W	–
TT13	24	21	1.14	4.7	Hoofprint	225°SW	T5
TT14	–	18		–	Hoofprint	–	T5
TT15	14	20	0.7	–	Hoofprint	247°SW	T6
TT16	14	18	0.78	–	Hoofprint	–	–
TTS17	26	27	0.96	–	Circular	–	–
TTS18	–	–		–	Circular	233°SW	–
MTS1	15.7	10	1.57	–	Hoofprint	n.d	–
MTS2	15	14	1.07	–	Hoofprint	n.d	T1
MTS3	16.3	13.8	1.18	–	Hoofprint	n.d	T1
MTS4	11	12	0.92	–	Hoofprint	n.d	–
MTS5	10	11.3	0.89	–	Hoofprint	n.d	–
MTS6	14.5	11	1.32	–	Hoofprint	n.d	–
MTS7	13	12	1.08	–	Hoofprint	n.d	–
MTS8	14	11.6	1.21	–	Hoofprint	n.d	–
MTS9	14.2	11	1.29	–	Hoofprint	n.d	–
MTS10	10.8	11.6	0.93	–	Hoofprint	n.d	–
MTS11	10.2	13.3	0.77	–	Hoofprint	n.d	–
MTS12	14	11	1.27	–	Hoofprint	n.d	–
MTS13	14	12	1.17	–	Hoofprint	n.d	–
MTS14	13	10	1.3	–	Hoofprint	n.d	–
MTS15	14	11	1.27	–	Hoofprint	n.d	–
MTS16	17.8	15.7	1.13	–	Hoofprint	n.d	–
MTS17	15	11	1.36	–	Hoofprint	n.d	–

The track length/width ratio and orientation patterns were examined by plotting, in bivariate and boxplot graphs, and in a rose diagram, respectively, the information of individual tracks (Fig. 3).

The storm surge of the spring 2020 exposed for a short period large area of the MTS, cleaning over 1.5 m of beach. Since the outcrop is located in the intertidal zone of Matalascañas beach, i.e., an area affected by intense tidal gradients, data recovery was limited in time and mainly consisted of taking photographs for photogrammetry and making casts for some of the tracks. The recognized tracks and trackways have been grouped in four morphotypes for mammals and three for birds. Likewise, insect traces have been recognized, as well as rhizoliths.

### Photogrammetry

Digital photogrammetry and GIS techniques have been used to produce safeguard records and highlight the morphological features of some of the tracks in both the TTS and the MTS. *Bovinichnus uripeda* holotype and paratypes were interpreted and described based on the field observations, measurements and analysis of the digital 3D models. Photos were made with a Canon PowerShot SX5OHS camera with a zoom lens 50xls 4.3–215 mm 1: 3.4–6.5 VSM. The photogrammetric models were processed using the software Meshroom 2021.1.0^77^ and ODM 2.1.0^78^, post-processed, analysed and highlighted in Meshlab v2020.12^79^ and CloudCompare v2.11.0^80^. The results obtained enabled to highlight with false colors the 3D morphology of the tracks^81,82^, making visual interpretation more effective.

## Supplementary Information

Supplementary Information.

## Data Availability

All data generated or analysed during this study are included in this published article and its supplementary information files.
