# Supplementary material for: Aurochs roamed along the SW coast of Andalusia (Spain) during Late Pleistocene
Source: Sci Rep. 2022 Jun 14;12:9911. doi: 10.1038/s41598-022-14137-6 (PMC9198092; doi:10.1038/s41598-022-14137-6)
Supplement: Supplementary file 1 — Supplementary Information. [file 41598_2022_14137_MOESM1_ESM.docx]

Supplementary material I

**Other tracks in the Cape Trafalgar eolianite**

Possible hominin track in cross section?

In the trampled surface IV, together with large hoofprints attributable to aurochs, occurs the cross section of a track produced by a flexible foot measuring 307 mm in length. According to the composite morphology of the track it can be interpreted as divided in three parts (Fig. S1a, b, c), recalling the human foot anatomy, posture and kinematics: (1) the deepest part corresponding to a rounded heel impression; (2) a convex medial part, with the shallowest impact depth, related to a dorsoflexed midfoot; (3) forefoot with deeper impression of the metatarsophalangeal area. The track proportions of the three parts are human-like. The deepness of the heel impression relative to the metatarsophalangeal area is consistent with down slope progression where the footfall has maximal pressure in the heel rather than the hallux^1^. The convex-up wide fold of the deformable sand laminae in the undertrack of the midfoot area reveals the plantarflexion and adduction during the stance phase^2^. The shear stress exerted on the substrate when pushing off with the forefoot has folded the sediment backwards and beneath the longitudinal arch. Finally, the metatarsophalangeal area is robust and shows deformation of the sediment related to the strongly flexing of the toes (Fig. S1c).

If the track found in the trampled surface IV in cross section is really human, it could only have been produced by a Neanderthal. The age of the Trafalgar Cape eolianite is much older than the earliest evidences of modern humans in Iberian Peninsula, between ~43-38 ka cal BP^3,4,5^. The foot proportions of Neanderthals were considered to be modern human-like, though Neanderthal feet are more robust and have larger joint surfaces^6^. Stature-track length inference^7^ points to an unusual tall individual around 2 m, tough. A range of statures up to 189 cm were inferred for Neanderthals from a large track sample described in Le Rozel^8^, but track length was found to vary considerably with the sand substrate conditions. The footprint may have been enlarged by the deformational motion of the foot over the sand substrate (Fig. S1b) but, nonetheless, it would need to be attributed to a very tall Neanderthal male.

The possible hominin track from Cape Trafalgar, fitting most of the criteria defined for hominin tracks in cross-section^9^, is together with Late Pleistocene (~90 ka) hominin tracks found in Brenton-on-Sea site on the Cape south coast at South Africa^10^, one of the first attempts to compare a fossil track in cross section with the human foot dynamics.

Elephant track in cross section

An isolated, large bowl-shaped track can be found in the trampled surface III together (Fig. S1d) with hoof prints. The true track (plantar-contact imprint) is 650 mm in length and 270 mm deep, with an undertrack of 890 mm resulting from the deformation of the underlying laminae (Fig. S1e). Microfolds and thrust faults underlie and surround the track resulting from the rotational movement produced by a stiff plantar surface during the strain process of foot-on-to-foot-off (Fig. S1f). Three phases of continuous deformation can be identified in the shaping of the undertrack^11^: (1) distal compression below the track-makers’s toes resulting in the shallower and least steep track wall; (2) rotation below the plantar surface creating a shear zone under the true track; (3) thrust faulting as the plantar load is released proximally. The resulting concave shaft was infilled conformably by subsequent sedimentation for a length of 440 mm (Fig. S1e). Proboscidean track soft-sediment deformation in cross section has been described in Late Pleistocene coastal deposits and, in most cases, related with tracks in bedding planes with the morphology of *Proboscipeda panfamilia* McNeil et al.^11,12,13,14,15,16,17^. In the SW Iberia these very large tracks could only have been produced by the straight-tusked elephant *Palaeoloxodon antiquus* (Falconer & Cautley)^12,17,18,19^*.* With a shoulder height of over 4 m, only a *P. antiquus* old bull could have produced one of the largest elephantine tracks ever described in the fossil record.

**Ichnogenus** *Pecoripeda* Vialov, 1965

*Pecoripeda* isp.

Description: Artiodactyl didactyl track composed by a *manus-pes* couple, of elongate wedge shape (Figs. S1g, h). The III and IV hoofprints are always distinct, with axial surfaces, separated by a continuous interdigital ridge. Hooves broadest near the heel, tapering to an apex of angular (hind print) and convergent, or sharply parabolic outline (fore print) and divergent, pointing forward^20^. Manus larger than pes, with 80 mm and 60 mm in length, respectively (Fig. S1h), and cleaves wider and divergent; *pes* with parallel, slightly curved hoofs with pointed tips.

Remarks: This track was found paired as *manus/pes* couple, the two tracks partially overprinting each other (Fig. S1g), which seems to be the case in *Pecoripeda*^18^. Roe deer tracks typically show a length of 40-50 mm and a shape close to the described *Pecoripeda* isp. However, roe deer tracks are slightly smaller and rounded at the back, with a division of the hooves at the front, producing a more heart-shaped trac^21^. Cleaves are narrow, with a pointed shape. Roe deer foreprints show their characteristic open cleaves and rear cleaves appear close together^22^. Domestic goat hooves are rounded at the ends and narrower at the front than the back. The cleave hoofs are often widely splayed and the individual cleave is convex on the outside and concave on the inside; dew claws do not leave imprints^22^. Domestic sheep prints can resemble roe deer’s, including in size, but are broad and more rectangular, with distinct, rounded cleave tips. Therefore, the *Pecoripeda* isp. tracks from Cape Trafalgar can be attributed to roe deer but a caprine cannot be dismissed.


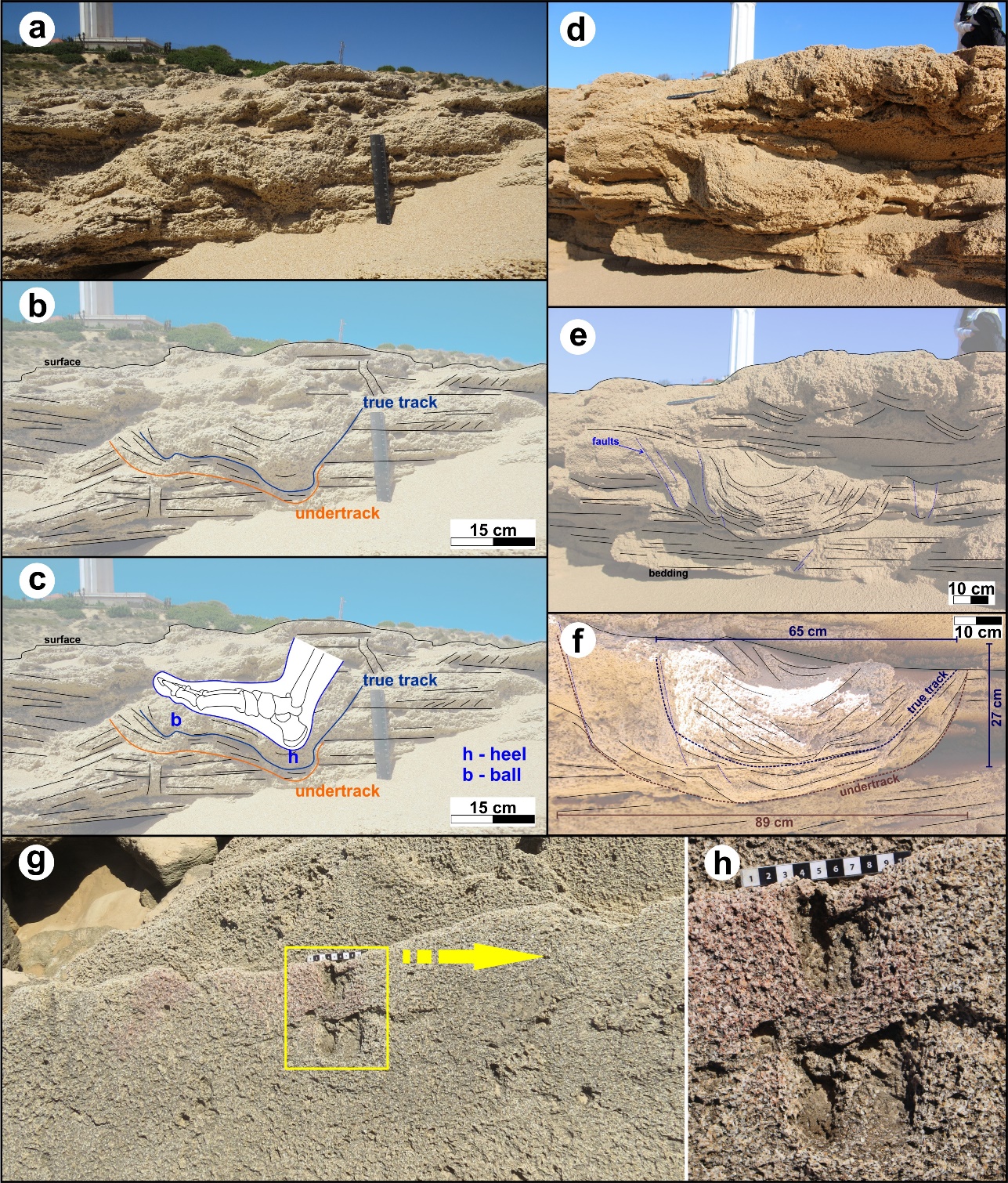
Fig S1. Other tracks found in the eolianite from the Cape Trafalgar. a-c) Track interpreted as hominin in section with the indication of lamina disruption and development of an undertrack below the filled true track (in b); foot dynamics interpreted in c; d-f) Large track that can only be attributed to a large elephantine: interpretation of the sediment deformation in e and the filling of the true track in f (measurements for the true and undertrack provided); g) *Pecoripeda* isp. couple where the well splayed foreprint is deformed by the narrow, pointed shaped hindprint (detailed in h). Figures b to f have been drawn with CorelDraw X7 (https://www.coreldraw.com/la/).

References

1. D’Août, K., Meirt, L., Van Ghelowe, B., De Clerq, D. & Aerts, P. Experimentally generated footprints in sand: analysis and consequences for the interpretation of fossil and forensic footprints. *Am. J. Phys. Anthrop*. **141**, 515-525 (2010).

2. Holowka, N. B., O’Neill, M. C., Thompson, N. E. & Dernes, B. Chimpanzee and human midfoot motion during bipedal walking and the evolution of the longitudinal arch of the foot. J. Hum. Evol. 104, 23-31 (2017).

3. Wood, R. et al. The chronology of the earliest Upper Palaeolithic in northern Iberia: new insights from l’Arbreda, Labeko Koba and La Viña. J. Hum. Evol. 69, 91-109 (2014).

4. Marín-Arroyo, A. B. et al. Chronological reassessment of the Middle to Upper Paleolithic transition and Early Upper Paleolithic cultures in Cantabrian Spain. PLoS ONE 13(4), e0194708 (2018).

5. Alcaraz-Castaño, M., Alcolea-González, J. J., Andrés-Sáez, J. A., Luque, L., Pérez-Díaz, S., Piqué, R., Ruiz-Alonso, M., Weniger, G.-C. & Yravedra, J. First modern human settlement recorded in the Iberian hinterland occurred during Heinrich Stadial 2 within harsh environmental conditions. Sci. Rep. 11:15161 doi: 10.1038/s41598-021.94408-w (2021).

6. DeSilva, J., McNutt, E., Benoit, J. & Zipfel, B. One small step: A review of Plio-Pleistocene hominin foot evolution. Am. J. Phys. Anthropol. 1-78 doi: 10.1002/ajpa.23750 (2021).

7. Mietto, P., Avanzini, M. & Rolandi, G. Palaeontology: human footprints in Pleistocene volcanic ash. Nature 422, 133 (2003).

8. Duveau, J., Berillon, G., Verna, C., Laisné, G. & Cliquet, D. The composition of a Neanderthal social group revealed by the hominin footprints at Le Rozel (Normandy, France). PNAS, doi/10.10.1073/pnas.1901789116 (2019).

9. Helm, C. W., Lockley, M. G., Cole. K., Noakes, T. D. & McCrea, R. T. Hominin tracks in southern Africa: A review and an approach to identification. *Palaeont. Africana* **53**, 81–96 (2019).

10. Helm, C. W., McCrea, R. T., Cawthra, H. C., Cowling, R. M., Lockley, M. G., Marean, C. W., Thesen, G. H. H., Pigeon, T.& Hattingh, S. A new Pleistocene hominin tracksite from the Cape south coast, South Africa. Scientific Reports. Online at: www.nature.com/articles/s41598-018-22059-5 (2018).

11. Bennett, M. R., Bustos, D., Belvedere, M., Martinez, P., Reynolds, S. C. & Urban, T. Soft-sediment deformation below mammoth tracks at White Sands National Monument (New Mexico) with implications for biomechanical inferences from tracks. *Palaeog. Palaeocl. Palaeoecol*. **527**, 25-38 (2019).

12. Neto de Carvalho, C., Figueiredo, S. & Belo, J. Vertebrate tracks and trackways from the Pleistocene eolianites of SW Portugal. *Comun. Geol.* **103** (I), 101-116 (2016).

13. Muñiz, F., Cáceres, L. M., Rodríguez-Vidal, J., Neto de Carvalho, C., Belo, J., Finlayson, C., Finlayson, G., Finlayson, S., Izquierdo, T., Abad, M., Jiménez-Espejo, F. J., Sugisaki, S., Gómez, P. & Ruiz, F. Following the last Neanderthals: Mammal tracks in Late Pleistocene coastal dunes of Gibraltar (S Iberian Peninsula). *Quat. Sci. Rev*. **217**, 297-309 (2019).

14. Lea, P. D. Vertebrate tracks in Pleistocene eolian sand-sheet deposits of Alaska. *Quat. Res*. **45**, 225-240 (1996).

15. Milàn, J., Theodorou, G., Loope, D. B., Panayides, I., Clemmensen, L.B. & Gkioni, M. Vertebrate tracks in Late Pleistocene-Early Holocene (?) carbonate aeolianites, Paphos, Cyprus. *Ann. Soc. Geol. Pol.* **85**, 507-514.

16. Neto de Carvalho, C., Figueiredo, S., Muñiz, F., Belo, J., Cunha, P. P., Baucon, A., Cáceres, L. M. & Rodríguez Vidal, J. Tracking the last elephants in Europe during the Würm Pleniglacial: the importance of the Late Pleistocene aeolianite record in SW Iberia. Ichnos **27**(3), 352-360 (2020). DOI: 10.1080/10420940.2020.1744586.

17. Helm, C.W., Lockley, M.G., Moolman, L., Cawthra, H.C., De Vynck, J.C., Dixon, M. G., Stear, W. & Thesen, G.H.H. Morphology of Pleistocene elephant tracks on South Africa’s Cape south coast and probable elephant trunk-drag impressions. *Quat. Res.*, 1-15 (2021) DOI: <https://doi.org/10.1017/qua.2021.32>.

18. Neto de Carvalho, C. Vertebrate tracksites from the Mid-Late Pleistocene eolianites of Portugal: the first record of elephant tracks in Europe. *Geol. Quart.* **53**(4), 407-414 (2009).

19. Neto de Carvalho, C., Belaústegui, Z., Toscano, A., Muñiz, F., Belo, J., Galán, J. M., Gómez, P., Cáceres, L. M., Rodríguez-Vidal, J., Cunha, P .P., Cachão, M., Ruiz, F., Ramirez-Cruzado, S., Giles-Gúzman, F., Finlayson, G., Finlayson, S. & Finlayson, C. First tracks of newborn straight‑tusked elephants (*Palaeoloxodon antiquus*). *Sci. Rep*., **11**:17311 (2021), <https://doi.org/10.1038/s41598-021-96754-1>.

20. Sarjeant, W. A. S. & Langston Jr., W. Vertebrate footprints and invertebrate traces from the Chadronian (Late Eocene) of Trans-Pecos, *Texas. Mem. Mus. Bull.* **36**, 1-86 (1994).

21. Milàn, J., Clemmensen, L. B., Buchardt, B. & Noe-Nygaard, N. A Late Holocene tracksite in the Lodbjerg dune system, Northwest Jylland, Denmark in *Cenozoic Vertebrate Tracks and Traces* (eds. Lucas, S., Spielmann, S. & Lockley, M. G.) 241-250 (N. Mex. Mus. Nat. Hist. Sc. Bull. 42, 2007).

22. Bang, P. & Dahlstrøm, P*. Animal Tracks and Signs.* Oxford University Press, UK (2001).
